# A conceptual model of urgent care sense-making and help-seeking: a qualitative interview study of urgent care users in England

**DOI:** 10.1186/s12913-019-4332-6

**Published:** 2019-07-12

**Authors:** Joanne Turnbull, Catherine Pope, Jane Prichard, Gemma McKenna, Anne Rogers

**Affiliations:** 1 School of Health Sciences, Faculty of Environmental and Life Sciences, University of Southampton, Southampton, SO17 1BJ UK; 2 NIHR CLAHRC Wessex, University of Southampton, Southampton, SO17 1BJ UK

**Keywords:** Urgent care, Patient work, Help-seeking, Sense-making, Qualitative methods, Healthcare utilization, Healthcare service

## Abstract

**Background:**

Theoretical models have sought to comprehend and conceptualise how people seek help from health professionals but it is unclear if such models apply to urgent care. Much previous research does not explain the complex interactions that influence how people make sense of urgent care and how this shapes service use. This paper aims to conceptualise the complexity of sense-making and help-seeking behaviour in peoples’ everyday evaluations of when and how to access modern urgent care provision.

**Methods:**

This study comprised longitudinal semi-structured interviews undertaken in the South of England. We purposively sampled participants 75+, 18–26 years, and from East/Central Europe (sub-sample of 41 received a second interview at + 6–12 months). Framework analysis was thematic and comparative.

**Results:**

The amount and nature of the effort (work) undertaken to make sense of urgent care was an overarching theme of the analysis. We distinguished three distinct types of work: *illness* work, *moral* work and *navigation* work. These take place at an individual level but are also shared or delegated across social networks and shaped by social context and time. We have developed a conceptual model that shows how people make sense of urgent care through work which then influences help-seeking decisions and action.

**Conclusions:**

There are important intersections between individual work and their social networks, further shaped by social context and time, to influence help-seeking. Recognising different, hidden or additional work for some groups may help design and configure services to support patient work in understanding and navigating urgent care.

**Electronic supplementary material:**

The online version of this article (10.1186/s12913-019-4332-6) contains supplementary material, which is available to authorized users.

## Background

The health services research literature includes now classic studies that attempted to conceptualise how people seek help from health professionals [1–3]. Theorising about help-seeking has, in the past, focused on utilisation behaviour for specific conditions (e.g. mental health [4]; long term conditions [5]), lay referral networks [6]; or organisational factors [6, 7]. In the past these have been used to inform help-seeking for urgent and emergency care. These explanations predate the expansion of urgent care and the provision of a range of expanded services for unscheduled contact and also the activity that lies at the interface between lay and formal systems of healthcare. In this paper we develop a model which builds on earlier literature to conceptualize help-seeking as a social process, one that entails different kinds of work, and which is grounded in the temporal, spatial and social contexts people occupy.

Urgent care is typically defined as healthcare that is needed for a condition that requires prompt attention (‘same day’ or within 24 h) but is not a life-threatening emergency. These services are designed to assess and manage unscheduled conditions, which often arise outside core office hours [8, 9]. NHS (National Health Service) urgent and emergency services are free of charge in the United Kingdom (UK). A range of urgent care services is available in England alongside emergency departments and the 999 emergency ambulance service. These include general practice out-of-hours services, a telephone-based triage non-emergency service that can assess and refer patients to different services (‘NHS 111’), walk-in centres, and minor injuries units. This expansion in service provision arises partly in response to consumerism [10, 11] underpinned by rhetorics of patient choice [12, 13]. A further policy imperative has been an aspiration to use urgent care to divert people away from overcrowded emergency services and to encourage greater use of self-care [8]. The impact of these structural changes has created an increasingly complex care landscape characterised by fragmentation and blurred boundaries between services [14]. In the face of this complexity, there is a need to understand peoples’ help-seeking behaviours and the work that they do to make sense of urgent care.

Research about help-seeking for urgent and/or emergency care using surveys and qualitative interview methods [15, 16] has shown that people seek urgent care about symptoms that are perceived to be severe, unusual, worsening, or causing pain [17–19]. Users make contact for medical care and advice, and to seek reassurance [20]. Much research reflects a pre-occupation with the ‘inappropriate’ use of emergency services [21, 22], often pointing out that people find it difficult to judge ‘appropriate’ contact and worry about using services unnecessarily [19, 23]. In a situation where contact with a service has yet to be made, appropriateness is negotiated in patient and professional interactions [24] and assumes a post-hoc attributed status [25]. While help-seeking is influenced by previous experiences and perceptions of accessibility [16, 26], several studies suggest that people occupy a liminal space, not knowing where to go [17, 26, 27] in which they consider and have to eliminate different, uncertain options. For example, people may use emergency department services when general practice is not available [28], or to access more ‘specialist’ care [16], or because the emergency department offers shorter waiting times and ease of access [26, 29]. Additionally, patients are increasingly using online information which may also shape decision making and help-seeking behaviour [30]. Many studies describe and categorise reasons for help-seeking but do not explain the complex interactions that influence why and how people process information and build understandings to make sense of urgent and emergency care and how, this in turn, shapes how they navigate and use services. This paper presents new data from a large qualitative interview study of urgent care users and proposes a conceptual model that attempts to capture the complex processes at play in peoples’ everyday evaluations of if, when, and how, to access urgent or emergency services.

### Theoretical background

Many factors have been shown to influence service utilization [31]. Theories of cognition, decision making and learning, drawn from social psychology focus attention on individual beliefs as triggers to help-seeking. The Health Belief Model, for example, describes psychological and motivational determinants of health service use, where cues to action (e.g. pain) and the readiness to act may be modified by individual and demographic characteristics such as gender, personality and social class [32], Andersen and colleagues [3, 33] showed how service use was influenced by individual level predisposing factors (e.g. health beliefs, age, education, social position) but also by community and enabling resources (e.g. income, access to transport). While this work showed that individual factors were associated with particular service use outcomes it placed little emphasis on social networks and social interactions [25]. While Andersen (1995) [34] suggests that these concepts broadly ‘fit into the social structure component’ of the model, they are not clearly explicated with the conceptual framework.

Work by medical sociologists has attempted to augment the understanding of help-seeking as a social process [1, 2, 35, 36] examining how and when help is sought, rather than service utilization per se [2]. Early work by Freidson (1961) [36] showed how lay referral networks influenced help-seeking behaviour. However, much of this work still presented help-seeking as the outcome of rational decision-making. Later work by Pescosolido (1992) [35] challenged this, presenting help-seeking as a more dynamic and contingent process in which socially constructed interactions and networks, and context influenced help-seeking.

More recently, Wyke et al. (2013) [37] have argued that models of illness behaviour have been developed in ‘disciplinary silos’. Existing theories and conceptualisations typically provide a set of psychological and/or social determinants that can be enrolled to explain urgent care help-seeking, but they offer less insight about the work that people do to make sense of their care needs, and how they assess the care options available to them at different points in time and illness trajectory.

Our paper attempts to re-focus attention on the ‘work’ people do to make sense of urgent care. There are many theories that have conceptualised patient work e.g. [38, 39]. Previous theorising around patient work has tended to focus on chronic illness but it is a useful means of understanding patient help-seeking concerning urgent care, and we particularly draw on concepts of work by Corbin and Strauss (1985) [38]. We also enrol the concept of sense-making as developed by Weick (1995) [40]. Our aim is to explicate how people make sense of urgent care through the work that they do when they experience a health problem, and in turn how this drives action e.g. seeking help from services or choosing not to seek help. This enables us to avoid simple binaries of ‘appropriate’ or ‘inappropriate’ service use and understand the processes and effort entailed in thinking (sense making) and acting (help-seeking). Our paper distinguishes three types of work patients do to make sense of their urgent care needs and services and we explore how social networks, contexts and time influence urgent care help-seeking.

## Methods

A typology of urgent care work and a conceptual model of urgent care sense-making and help-seeking was developed from analysis of serial semi-structured interviews that explored perspectives, experiences and decision making around urgent care. Ethical approval was granted from the NHS Health Research Authority (16/EM/0329).

### Recruitment

Participants were drawn from four counties in Southern England. We purposively sampled to represent potential differences in urgent care need, and socioeconomic and demographic characteristics: people aged 75+ years, and those aged 18–26 years were chosen to reflect populations with known high use of emergency care and a third group, people from East and Central European communities, was chosen to capture the experiences of recent migrant populations. Whilst there are other groups that are known high users of services, such as parents of young children [26, 41, 42] the populations in our study were chosen because they are groups for whom we lack evidence about help-seeking and decision making around health service use. We recruited people using a combination of community-based and local media advertising, and from those attending NHS urgent and emergency care services. Interested participants were either sent an information pack or provided with this by a research nurse. To encourage participation a £15 gift voucher was given for each interview.

We conducted first interviews with 100 people, 66 of whom were female. Seven interviews were in pairs (usually older couples where both decided to participate) and all were conducted between September 2016 and July 2017. Participants were invited to take part in second interviews and 41 people agreed to take part in a second interview conducted between June 2017 and November 2018. Table 1 shows the number of participants in each of the three targeted population groups.

**Table 1:** Number of interview participants by population group

Interview	Population group	Number of participants
Interview 1	East European	18
	Older (75+ years)	43
	Younger (18–26 years)	39
	Total	100
Interview 2	East European	12
	Older (75+ years)	19
	Younger (18–26 years)	10
	Total	41

### Data collection

First interviews explored peoples’ understandings of urgent care, how they distinguished between routine, urgent and emergency care needs, and their knowledge of available services. The second interview explored issues raised in more detail and focussed particularly on recent experiences and decision making about urgent or emergency care help-seeking. Topic guides were used (See Additional file 1 – Interview topic guides), informed by the literature and by citizens panels conducted in the wider project [14]. Interviews were face-to-face, carried out by two female members of the research team (GM and JT] and lasted between 35 and 90 min. Most interviews were conducted at participants’ homes, but a small number were conducted at University offices or other premises. The interviews were digitally recorded, with consent, and transcribed verbatim as anonymised documents for analysis.

### Analysis and developing the model

We used a thematic analytical approach, broadly following the stages described by Braun and Clarke (2006) [43]. The research team (JT, CP, JP, GM, AR) initially read and open coded a sample of transcripts independently, then discussed emerging codes to form the basis for a coding scheme which was refined and applied to all transcripts. The team worked together to interpret data, building emergent themes and developing narrative and interpretive summaries, using Atlas.Ti to manage and access data. In the later stages we drew on the Framework approach [44] creating matrices and charts to aid comparative analysis. We also created typologies to map our interpretations and begin sketching the relationships and connections in the model, referring back to the data and thematic analysis throughout this process. We continued to use comparative analysis to identify factors that were common or contradictory in different care contexts and in different populations. From this work we were able to test emerging hypotheses about how sense-making and help-seeking related to each other, providing the framework for the conceptual model presented in this paper.

## Results

The amount, type and nature of the work undertaken to make sense of urgent care was an overarching theme identified early in the analysis. We distinguished three related, but distinct, types of work: *illness*, *moral* and *navigation* work (Table 2). These are discussed in turn in the next sections of the paper. We show how work takes place at an individual level but may also be shared or delegated across social networks and shaped by social context and time. We use these ideas to elaborate a conceptual model that shows how people make sense of urgent care through work which in turn influences help-seeking decisions.

**Table 2:** Typology of urgent care work

Concept of work	Individual level	Social network level	Nature of the work
Illness work	Assess and manage symptoms, regimens, and risk, and actions associated with these	Assess and manage symptoms, regimens, and risk, and actions associated with these across social network members	• Interpret, manage, evaluate symptoms and risk of symptoms • Identify what is being sought from a service • Decide if help is needed and/or level of care required
Moral work	Assess and legitimate ‘appropriate’ service choices	Assess and legitimate service choices in comparison to others and influenced by others	• Decide what is appropriate use • Construct self as credible, responsible and appropriate service user • Balance moral positioning against health risk
Navigation work	Assess services available (choose) and decide which to access (use)	Assess services available (choose) and decide which to access (use) informed by social network	• Know about, and choose, services, facilities and resources available at different times of day • Decide the most acceptable or convenient choice

### The work of making sense of urgent care

#### Illness work

Our theme of ‘illness work’ draws on previous theorising about symptom management [1, 2, 38]. People make sense of illness by interpreting the severity of symptoms, managing physical and their psychological state, assessing risks, and making decisions about accessing services. Participants reported that symptoms that were ‘sudden’, unusual, ‘serious’, or interfered with daily life (e.g. impaired mobility) were likely to prompt help-seeking. This finding reflects the extensive literature and we will not rehearse this here. Instead, we focus on some distinctive prompts to urgent care help-seeking, related to *uncertainty* and *social network involvement*. Uncertainty about symptoms often provoked anxiety. Those reporting lower levels of anxiety tended to seek reassurance from urgent care services like NHS 111, but those who were more worried used emergency care.

***P19:*** *I didn’t know what else to do at the time because I was in a state. Well not panic, but I was highly stressed. I thought ‘I don’t know what to do’ so I just dialled 999 [ambulance service].* (Older).

Managing uncertainty about symptoms entailed ‘risk assessment and management work’. Participants sought reassurance from health professionals or members of lay networks to ‘*be on the safe side*’ and manage potential risks. NHS 111 was often the first port of call, particularly for younger and East European participants. Many considered the perceived limitations of their own expertise when interpreting and managing symptoms and so drew on others in their social network to sanction decisions (e.g. about whether to contact a particular health service).

***P66:*** *I called my dad ... ‘Dad … can you bring a first aid kit and just give me an opinion on whether you think this is a bad enough cut?’ Because I just... I didn’t know...* (Younger).

Those who felt responsible for the health of others (children, or a partner) and where the frequency of interaction was high (e.g. living in the same household) described social networks exerting a strong influence on this illness work.

***P52:*** *Well it’d be you, wouldn’t it [husband]? Then [our] daughter and then the GP [general practitioner].* (Older).

***P53:*** *I think the most important one would be my mum, as well as my closest friends. The least, would be work. Yes, [and] the internet.* (East European).

In the case of Eastern European participants, their migrant status resulted in them having smaller local networks and as a result they often connected with family and close friends in their home countries by telephone. Weaker ties such as acquaintances and neighbours played a significant role in older peoples’ help-seeking because they lived close by and were often at home during the day.

***P93:*** *The neighbours are brilliant. They are so important as they are invariably there. Or I could phone our really good friends who are a 10-min drive away…then the GP [general practitioner]. If I needed my son or daughter for anything … well, yes, if I needed them to come, I know they would come.* (Older, married carer with children far away).

In line with previous research people in their networks who were ‘experts’ by profession or experience were particularly valued. Younger participants, whilst they often discussed illness with friends, often did not appear to trust their opinions. Instead parents were viewed as knowledgeable, a more credible source of help.

***P54:*** *I can definitely put my husband and mother [as sources of support] … She’s a Doctor … a paediatrician.* (East European).

***P65:*** *My mum was really supportive. Friends…a little less so, because … just, especially at that age* [18]*, I don’t think anybody really, had much of a clue. Couldn’t empathise properly with what I was going through … ‘oh, he’s always ill’ … ‘attention and whatever’.* (Younger).

Seeking such advice had to be balanced against worrying others. This was most common in parent-child relationships; younger people did not want their parents to worry and vice versa. In these circumstances participants reported coping on their own, using online resources, or drawing on weaker ties.

***P54:*** *I may use my mum sometimes but I just don’t really want to bother her because she’s just going to be so worried. I would probably prefer to do … online symptom checks.* (East European).

#### Moral work

The term ‘moral work’ describes work undertaken to present as an appropriate, legitimate or responsible user of healthcare - ‘a credible patient’ [15, 45]. There was a tension between a service user’s desire to represent themselves as responsible citizens (e.g. confident in their ability to self-care and make rational judgements) and thus avoid being labelled as a ‘time waster’, and a desire to delegate illness work to healthcare professionals. The moral work involved is multifaceted, undertaking the moral responsibility of being a ‘good self-manager’ (taking responsibility for own health, and using knowledge to manage risks) [46] to enable ‘appropriate’ judgements about the nature of urgent symptoms. Service users weighed up the risk of harm against taking action. Across all groups, participants were keen to demonstrate their responsibility, providing accounts of when they had not sought help or examples of symptoms they considered trivial. Many described themselves as ‘copers’, who tolerated symptoms and performed self-care. Not accessing services was a sign of stoicism, or resilience, of which people were proud.

***P23:*** *I think we were brought up in that generation, at the beginning of the war, and you had to get on with life [*] *You just try not to bother people. I never go to the doctors, if I can help it [*] *They’re [the ambulance service] up to their eyes.* (Older).

Many service users acknowledged that emergency services experienced high and pressing demand. They were all too aware that accessing services ‘unnecessarily’ might waste scarce resources and deprive care from those ‘who really need it’. Fear of negative reactions from emergency care providers might push them to use urgent care, younger participants preferred to contact NHS 111 and older people used the pharmacists to avoid ‘bothering the doctor’.

***P57:*** *There would be an instinct in me [to use NHS 111] … I don’t want to make a fuss out of something that might not be a fuss, or I don’t want to annoy the doctor.* (Younger).

***P5:*** *It was pretty easy to make the decision to go to the pharmacy, so I wouldn’t have bothered the doctor or even the nurse.* (Older).

Moral work included efforts to compare and reference service use against that of ‘others’. Other people were designated as time wasters, and one’s own ‘responsible use of services’, or coping was contrasted with those who ‘*rush off to the doctors’*. P13 for example, disapproved of others but admitted using the emergency department for a more minor issue because she has ‘panicked’:

***P13:*** *People panic so much. They can have a little thing like ‘alright you’ve broken your arm, it’s going to hurt like hell, but it’s not a big deal, you’re not dying’ … You need to go to A&E [Accident & Emergency Department] when you are bleeding like severely, or something … fatal … Unfortunately, we were there for something that really was not quite an emergency … but I panicked.* (Younger).

Social network members supported moral work by sanctioning help-seeking and alleviating individual responsibility for decision making. Contact with services for less serious symptoms was sometimes attributed to the insistence of network members. Younger service users cited others (usually parents, but sometimes managers at work) as instrumental in help-seeking.

***P68:*** *I tend to play down a lot of how I’m feeling, because I don’t like going to the doctor and I don’t want to go to hospital, but my boss said ‘no, I think you need to call [NHS] 111, you’re clearly not right’.* (Younger).

#### Navigation work

‘Navigation work’ involved in identifying and making sense of the range of services on offer and how to access healthcare services. It also involves network navigation - identifying (from pre-existing relationships) who should be contacted to make decisions, prioritizing access to some members in the social network over others [47]. We have drawn on Penchansky and Thomas’s (1981) [48] dimensions of access to inform this theme. Service users made choices between what was available (e.g. staffing, resources, technology), accessible (the ease with which a health service can be physically reached), and ‘accommodating’ (e.g. convenient opening hours). This navigation was informed by present relationships but also past experience, knowledge and perceptions about illness and about services which recursively shaped future help-seeking [16, 25]. There was considerable confusion about when to access urgent care services. In contrast, there was greater confidence about the services provided in the emergency department which was often seen as a ‘one stop shop’ where additional specialist facilities (such as X-ray) could be accessed. Choosing to attend an emergency department maximised the chances that the facilities needed would be available – thus avoiding the risk of a potentially wasted journey.

***P70:*** *It’s a nuisance to get to the hospital because it is an hour away but once you are there…*

***Interviewer:*** *It’s all there?*

***P70*** *: At the hospital [laughs]*

***Interviewer:*** *So here in town, there are different services [urgent care] at different places…*

***P70****: Yes. And that’s a bit of a pain.* (Older)

However, the emergency department was avoided by those who perceived it as a busy or unpleasant environment. Urgent care was sometimes described as a more comfortable and less crowded environment.

***P5:*** *I prefer to go to one of the drop-in centres, rather than up to [ED] because ... it’s usually overflowing, isn’t it, with people waiting to be seen ... It’s a pleasanter experience, anyway, at [the Walk in Centre].* (Older).

One of the drivers of sense-making about service use was waiting time. Urgent care services were viewed as an available and more convenient than General Practice by all three groups, primarily because an appointment was not required. P1 disliked the lengthy waiting times at the walk-in centre but she used it when she was unable to obtain a General Practitioner appointment.

***P1:*** *It’s more difficult to go to walking-in centre because most times you wait … You fill in loads of forms and definitely it will take you at least two, three hours [*] *I think the walking-in centre is good if you can’t get your appointment at GP [General Practitioner].* (East European).

Decisions were based on efficiency and convenience rather than an assessment of severity or clinical need. The model of the patient as consumer was apparent in such accounts:

***P37:*** *If you think something is not all that wrong but you still need to go to A&E, having the luxury of picking and choosing the time, like, go in the early hours of the morning because that might be a bit emptier, you’ll be seen quicker.* (Younger).

***P46:*** *The A&E was very calm, they served us pretty quickly in, like, half an hour or so.* (Younger).

Some people looked up information on the internet or telephoned the emergency department to enquire about waiting time before travelling. When P67 did this, she was advised to attend later as it may be less busy, which potentially reinforced the use of the emergency department for something that ‘wasn’t that serious’:

***P67:*** *With the knee, I think Friday or Saturday night, we actually didn’t want to go to A&E because you obviously spend, sometimes a few hours and it wasn’t that serious [*] *So we just rang them up and said ‘what is the approximate waiting time?’ … and they say ‘it is busy but you can try later on’* (East European).

While some recognised that using the emergency department for more minor medical problems wasn’t the most appropriate choice several noted that difficulties of travelling justified their decision.

***P22:*** *I knew it wasn’t really the correct place. I wanted a walk-in centre but there isn’t a convenient one … There’s the one in [area name] but trying to get [there] is murder, and the other one, it’s so far away you could die on the way.* (Older).

The NHS 111 telephone service offered the benefits of avoiding unnecessary waiting, travel or an unpleasant environment and was attractive, especially to younger participants.

***P76:*** *111… you ring them up and they kind of assess you on the phone … you do not have to go all the way to hospital … talking on the phone beforehand [is] a better option because when you are in A&E you are around so many people who have different problems, a lot of it is people on drugs, or drunk … it’s a bit overwhelming* (Younger).

While much of our data suggested that navigation work was performed individually, some consulted relatives, friends and neighbours. Younger participants in particular, used the internet to seek out information from social media networks as well as family members to help navigate services.

***P40:*** *I tend to make my own decisions but sometimes I’ll ask my mum. But I know a lot of people … ask Facebook sort of thing.* (Younger).

Social networks provided information about different services and supported choices about service use:

***P28:*** *If you’re on your own, it’s different. I mean, if I hadn’t had [friend] to talk to, I wouldn’t have gone to A&E at that stage. I might have left it a bit later.* (Older).

***P7:*** *I’m lucky because I have backup around me, or even the neighbours. I mean, for my eyes, I talked to several people within the [street] … then there’s the U3A [University of the Third Age, an international movement for mainly retired members], [the] Choir and the Masons.* (Older).

Whilst close interpersonal ties were highly pertinent for East Europeans in illness work, their navigation work was dependent on having access to local knowledge of services. This put recent migrants at a disadvantage, as P2 explained, fragmented networks could make it difficult to navigate UK health services.

***P2:*** *The Polish community families, the new families, are quite fragmented. So it’s people who have got to know each other over here … normally, back in Poland, the community is very close … you tended to be born somewhere, find work, study around that area, and have family not far … Whereas here, I think people tend to panic a little bit … a young family, isolated from everybody. They don’t know who to go to, so go to A&E.* (East European).

### The relevance and influence of socio-temporal context

Sense-making was shaped by social context and time, and together these factors could facilitate or inhibit support-giving and help-seeking. Help-seeking could be facilitated for those who lived with a partner, owned a car, or who worked flexible hours or who found themselves in a domestic or work context where organizational arrangements and resources available differ. Those who lived alone, or relied on public transport often made different choices to those surrounded by other people with ready access to getting around. Time of day interacted with social context to influence the help available and how people felt about asking for help (for example, there is a greater reluctance to ask others for help at night). Participants often acknowledged the importance of the socio-temporal context in which illness was experienced, especially those who lived alone, or for health problems that occurred at night.

***P2****: [There is] the likelihood of me … over-stating … you know, exaggerating. Because it’s me, and I’m on my own. It’s me, me, me. Yes, especially at night things may seem, a lot more drastic than they really are.* (East European).

***P28:*** *I knew [a friend] would take me. But she had to come over from [different area] ... If somebody’s taking me... she’s got to get here, she’s got to get back afterwards.* (Older).

***P23:*** *My neighbour … I’ve only got to ring her and she was round … She is always there for me … But … I thought, ‘this is like 7, 7:30 at night, I’m not going to be able to cope with him [husband] on my own, [later] in the night, if it goes on like this’. I know … if I’d have rung [her neighbour] she can come around. But there’s something different about bothering them in the middle of the night to the daytime.* (Older).

Caring for others appeared to amplify the salience of moral and navigation work and factored into help-seeking practices:

***P1:*** *When you’ve got children [when husband was ill], should I take them with me [to healthcare service]? [*] *I don’t know if I could ask neighbours to come … because I never had to try that. But I can phone for example, my sister …* (East European).

Paid work also influenced help-seeking. For some, it meant accessing care that best fitted around working hours by attending a walk-in centre rather than making a General Practice appointment. This was a particularly common for East European and younger participants who were most likely to be negotiating healthcare in the context of paid employment, studying and family commitments.

***P32:*** *Monday is quite a busy day [for health services] because everyone waits the weekend … I work nine to five so I would probably try to do it out-of-hours if possible or over my lunch break.* (Younger).

Navigation work was more complex and tentative for those who relied on others for transport. Older participants and/or those with mobility difficulties, including people who were frail or recovering from illness or injury, noted that availability of transport, support and time of day influenced their choices.

***P55:*** *If it’s the weekend then I would contact them normally. But they’re not always around, you see? They’re all at work. You can’t rely on your family.* (Older).

***P23:*** *[The bus] stops at the bottom of the road … then it stops outside the hospital. It’s free for me because I’ve got a bus pass … If I can, I get there under my own steam. My neighbour really gets angry with me because I don’t ask for a lift … I said, ‘all the time I can do it, I will do it’.* (Older).

The potential difficulties of English as a second language was not a common theme in our data, in part because our data collection method required some proficiency in English. However, some East European participants noted that language could hinder navigation work. Services accessed by telephone presented more difficulties, as this participant noted.

***P47a:*** *Is there like option [with NHS 111] … to avoid all the questions? Because … obviously people with less understanding English they get confused as well.* (East European).

While our data does not represent other communities for whom language may be barrier, such as those with learning or communication difficulties, these are likely to shape choices in similar ways.

## Discussion

Our findings resonate with existing literature [24, 49] but illuminate the social processes relevant to urgent and emergency care help-seeking. We have shown that service users hold strong moral views and are highly sensitive to arguments about ‘inappropriate’ help-seeking in the emergency department. However, they often externalise these judgements such that moral rules are applied to others (e.g. characterising others as ‘time wasters’) [10, 23, 27]. We observed fewer moral judgements regarding the ‘misuse’ of urgent care services and this seems to reflect the dominance in public discourse about ‘overcrowded’ emergency departments and the idea that such services are sanctioned as needing to be available to all comers. We have also shown that people make choices influenced by what is accessible at a given time of day [25, 48]. Urgent care provision is variable, and there is inconsistency in provision across different areas. Waiting time is a strong factor in decision making. National Audit Office figures suggest that patients registered with general practices that are open fewer than 45 h per week attend the emergency department more often [50]. Road and transport links may further influence accessibility of some services [26] and our data suggests that proximity is temporal as well as geographical (e.g. the hospital may be ‘nearer’ at night because of car and motorway access). These temporal and spatial features are highly socially patterned: older people for example relied more on others for transport than other groups, recent East European migrants may have less knowledge of services in their locality and this will limit their choices.

**Fig. 1:**
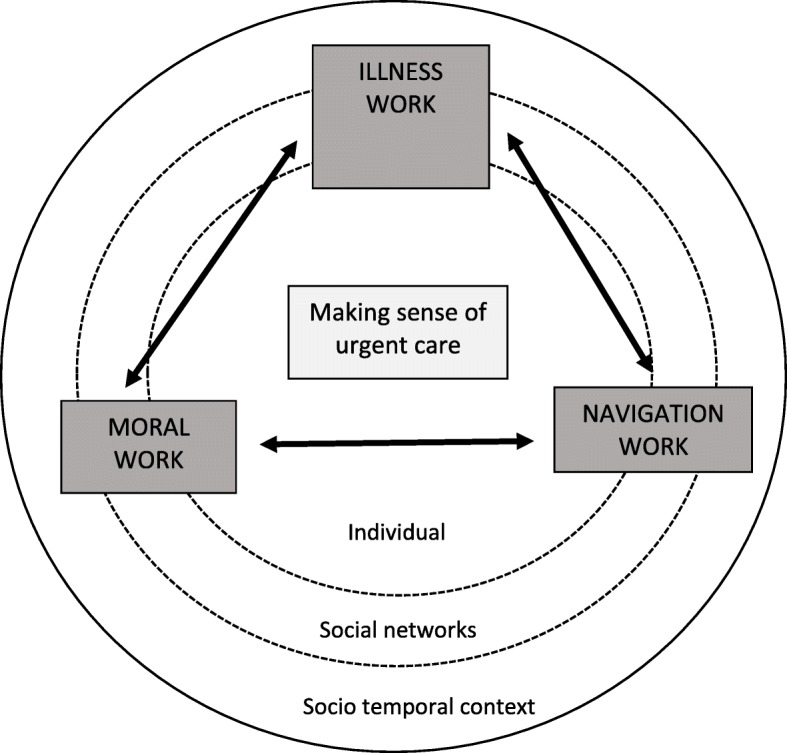
Model of urgent care sense-making and help-seeking

Our findings illuminated a typology of three types of work that determine how people make sense of, and seek help from, urgent care and illuminates the multi-factorial mechanisms underlying drivers to access urgent care. Our analysis showed that this work did not only occur at the individual level, it was clear that social network connections and the wider context and contingencies of everyday life also influenced sense-making and help seeking. For example, we have shown that navigating service use is a complex interplay between individuals and their social networks, but is also highly dependent on social context and time. Kin and friendship networks may support sense making and choices to attend particular services but this will vary at different times of the day. Social networks can provide practical resources (such as transport) to assist help seeking, but members of recent migrant communities, or an older person experiencing illness at night may not have access to this support. Reserves of social capital are thus pertinent to determining urgent care attendance despite the service itself being available to all comers. We have attempted to represent the relationships between the three types of work identified (shown in the boxes in Fig. 1), and the network and temporal contexts in a conceptual model. We suggest that urgent care sense-making and help-seeking might best be seen as a social process that requires the careful balancing of (sometimes competing) types of work within contexts (the latter represented by concentric circles). The ‘choice’ to attend an emergency department or an urgent care service requires illness work to operate in a way which discerns what is needed (e.g. individual symptom identification, social network reassurance), and work to legitimise a moral position (again, individual and social articulation of ‘my service use as appropriate’) as well as the individual ability and network resources to navigate the complex landscape of care. This complex interplay between work by individuals and their social networks to make sense of care, and take action, is seemingly contingent on socio-temporal context (i.e. a process which will vary across groups, settings and time).

### Strengths and limitations

We have drawn on, and extended, existing concepts of help-seeking and have applied these to acute rather than chronic illness. Data from our large interview sample allowed us to delineate a typology of the work entailed in sense making and help-seeking. This builds on the core concept of individual illness work, adding in moral and navigational work and extending the conceptualisation to emphasise the role of social networks and socio-temporal context. Previous research of this kind has often been limited by small sample sizes, and has tended to focus on particular determinants (e.g. waiting times or transport), and/or on specific patient groups. We have looked at how a large and diverse sample of service users make sense of, and use urgent care. We examined three specific population groups, determined not by the nature of their illness but by their demographic features. Elderly populations have been the focus of other studies [51] but younger and migrant populations have received less attention. Studying these three different populations allowed identification of group differences and a more holistic understanding of sense-making and the moderators of their decision-making processes.

We cannot be sure that the South of England fully reflects the full range of views and experiences across the UK and elsewhere. Whilst this setting is not the most socio-economically deprived when compared with other parts, the area includes pockets of deprivation and some areas are in the most deprived quintile nationally (e.g. parts of Portsmouth, Southampton, Reading), and contains areas that are in the most affluent categories (e.g. Wokingham, Aylesbury). The setting also includes major cities (such as Portsmouth, Southampton, Oxford) and a mix of urban and accessible, and more remote rural areas. It is possible that our three groups do not fully reflect the full range of views and experiences, for example, in the Eastern European group, those that participated had very good levels of spoken English and were well educated. It is not clear if we would have observed similar sense-making and help-seeking in a more diverse group, but it seems likely that lower levels of English language may lead to even greater difficulties in navigating urgent care. Similarly, our younger group were largely recruited through educational establishments and may thus have a higher level of education compared to the wider population. In addition the use of interviews enabled us to capture peoples’ self-reported accounts of service use, not actual use. To mitigate this the use of follow-up interviews provided some opportunity to probe accounts and explore encounters with health services that took place between interviews and this may have encouraged more reflexive insights about actual behaviours.

## Conclusions

Our conceptual model frames the interaction between thinking (sense-making) and action (help-seeking) and emphasizes the work implicated (for individuals and their social networks) in accessing urgent care. It shows how the individual and their social networks work to interpret illness, make moral judgements and navigate services. Traditionally policy and research has focussed on service use outcomes, from which we infer ‘wrong decisions’ and ‘inappropriate help-seeking’. Our model suggests that to change outcomes, there needs to be a change to the work people do, collectively and individually. This understanding of work may mean that policy and interventions focus less on blaming ‘incorrect’ sense-making and ‘inappropriate’ decision making, and begin to support patient work. People do not deliberately make ‘wrong’ help-seeking choices, these choices are a product of the work that they do. Recognising that different or additional work may be required for different groups (e.g. different age groups, migrant populations) can inform service design and signposting, but must be directed at the work these groups have to do. For example, some migrant groups will have no experience of non-hospital based urgent care. They need support to navigate this different care landscape and health and local authorities might need to consider making service ‘maps’ available in relevant languages. Advertising and health education campaigns could better reflect the social and temporal drivers that might push people towards particular services, for example acknowledging that sense making and help seeking are different at different times of the day.

At a structural level the impact of the frequent reconfiguration and extension of urgent and emergency care services on patient work should be considered. Patient-centred co-design methods, could be enrolled to better demarcate the routes from self-care, to primary, urgent and emergency care in ways that the public can understand. Recent UK policy has proposed a single multidisciplinary Clinical Assessment Service within integrated urgent care services ‘to provide specialist advice, treatment and referral’, and ‘encompass both physical and mental health’ [52]; this may support some of the work we have outlined. Beyond this there are structural changes, such as standardisation of Urgent Treatment Centres opening hours and facilities [52] that will support the work entailed in sense-making and help-seeking. Policy and provision has increasingly focused on signposting and standardizing urgent care services. Such approaches may help reduce the complexity of urgent care work that patients are expected to engage in. It is clear that changing peoples’ help-seeking (action) is contingent on changing the nature of urgent care work, to encourage better experiences of urgent care and more effective health care use.

## Additional file

Additional file1: Interview topic guides. (DOCX 21 kb)

## Data Availability

The datasets generated and/or analyzed during the current study are not publicly available. This is because participants did not consent for the data collected to be made public or shared with any other parties. Participants did consent to the use of anonymized quotes as used in the manuscript.

## Abbreviations

‘999’: ‘999’ emergency ambulance service
A&E: Accident and Emergency Department
GP: General Practitioner
NHS: National Health Service
UK: United Kingdom
