# Supplementary material for: A conceptual model of urgent care sense-making and help-seeking: a qualitative interview study of urgent care users in England
Source: BMC Health Serv Res. 2019 Jul 12;19:481. doi: 10.1186/s12913-019-4332-6 (PMC6624886; doi:10.1186/s12913-019-4332-6)
Supplement: Supplementary file 1 — Interview topic guides. (DOCX 21 kb) [file 12913_2019_4332_MOESM1_ESM.docx]

**TOPIC GUIDE FOR SERVICE USER INTERVIEWS - INTERVIEW 1**

**General background / context - participant's health and social circumstances**

- Family context (guardian or carer) / history / work outside the home / friends and family
- General health / long-term condition / multiple conditions
- Location urban / rural

**Making sense of urgent care**

- What does ‘urgent care’ mean to you?
- What does ‘emergency care’ mean to you?
- What services do you view as ‘urgent care’ services?
- How do you distinguish between urgent care services and emergency care services?
- What would you describe as an ‘urgent care’ problem?
- How would you describe the similarities and differences between urgent and emergency care?
- How easy or difficult do you think it is to decide when to use urgent care?
- Why would you use an urgent care service? In what circumstances?
- Why do you think others use urgent care services? In what circumstances?

**Expectations, beliefs and knowledge about different urgent care services (111, WICs, MIUs)**

- Explore knowledge of services (e.g. what services are available to you locally? What do you think are the differences are e.g. an urgent care centre, a minor injuries unit and the 111 telephone service? Alternative avenues of urgent care; Do you know what to expect when you contact different sorts of urgent and emergency care services?
- Explore decision making (e.g. What do you think would be helpful to you in deciding how and when to use urgent care? Sources of advice or information about urgent and emergency care services (Lay networks; advertising/media; previous use)
- Explore perceptions about the quality of urgent care (e.g. Do you think urgent care services could be improved? If so, how?)
- Explore relationship between GP and urgent care
- Explore views about ‘other people’s’ use of urgent care
- Explore perceptions about what is ‘appropriate’ / responsible use of urgent care services

**Previous experiences of using urgent and emergency care**

- Explore previous experiences e.g. which services they have used,
- **How easy or difficult was it to access urgent care?**
- Contextualising the call: narrative account of background leading up to the call or attendance [final precipitating and triggering factors; **any self-care or triage**]
- What did you think the problem was?
- **How serious would you say the problem was? (quick onset of symptoms; anxiety levels)**
- Your story, your account of your contact, your experiences of using urgent or emergency care in recent years - timeline of symptoms/decisions, **your satisfaction with the care received (including self-care on discharge)**
- Alternatives to OOHs? Consider any other health service?
- How has this compared to previous experiences of illness?
- Reflections after the treatment episode: thoughts about future care.

### TOPIC GUIDE FOR SERVICE USER INTERVIEWS - INTERVIEW 2

**Reflecting since the previous interview on a specific experience**

- Have you accessed an urgent or emergency care service since the previous interview?
- If so, explore your story, your account, your experiences of using urgent or emergency care
- Reflect on specific instances of using a service, the circumstances surrounding these and the factors which influenced these decisions
- Reflect on times when you did not use urgent or emergency care, and on what influenced decisions not to use these services

**Making choices**

- If you had a healthcare problem in the evening that couldn’t wait until morning how would you decide what to do?
- If someone you were caring for had a healthcare problem in the evening that couldn’t wait until the morning, how would you decide what to do?
- How do you choose what service to use?

**Burden of treatment (the ‘workload’ of healthcare’) - navigating and accessing care**

- What are your experiences of availability of services locally?
  - opening hours; distance/transport; gatekeepers; negotiation; ‘joined-up ness’;
- What do you think are the disadvantages and benefits of using particular services?
- How easy or difficult is it to access urgent or emergency health services? How convenient are different sorts of services?
- What is it like to live here? Does where you live affect the decisions you make?
- What does it require of you to make contact or attend an urgent or emergency service?
- How easy or difficult is it to decide what service to use?
- In caring for your health, do you get support from other people? Who? What kinds of things do they do to help you? Has your health care ever created tension between you and other people?
- For some people, the personal work of caring for their health condition can be emotionally challenging? Is this true for you? (Explore where participants care for others)
- For some people, the personal work of caring for their health condition can be financially challenging? Is this true for you? (prompts - telephone, prescriptions, physical / medicinal aids).
